# India Fighting COVID-19: Experiences and Lessons Learned From the Successful Kerala and Bhilwara Models

**DOI:** 10.1017/dmp.2021.115

**Published:** 2021-04-19

**Authors:** Rishita Chandra, Smita Sinha

**Affiliations:** Department of Community and Family Medicine, All India Institute of Medical Sciences, Rishikesh, Uttarakhand, India

**Keywords:** COVID-19, severe acute respiratory syndrome coronavirus 2, containment field model

## Abstract

The COVID-19 pandemic marks its emergence in China in December 2019. India reported its first case on January 30th 2020 which happened to be epidemiologically linked to China. On March 24, India went into nationwide lockdown. The number of cases increased in the country and a few hotspots were identified. Cluster containment strategy seemed to be effective in containing the disease and breaking the chain of transmission. Two models (Kerala and Bhilwara) emerged as a lesson for other states. Kerala government implemented a “triple-lock containment strategy” and Bhilwara district administration followed “all down curfew” with massive sample testing. The experiences from these successful field models can be implemented in other districts and states to flatten the COVID-19 curve.

## Introduction

The global coronavirus disease (COVID-19) pandemic has affected several countries across the globe. The first death from COVID-19 was reported by China on January 11, 2020.^1^ On January 30, 2020, India reported the first domestic case of COVID-19, which happened to be epidemiologically linked to China. The time-lapse between the first confirmed case and completing a century in India was almost 3 months, which again quadrupled over the next 10 days. The period during which almost every nation started to get a surge in COVID-19 positive cases, the 2 field models for containment of the disease spread emerged as examples from the state of Kerala and Bhilwara District of Rajasthan, India. The specific strategy and effective implementation with strict monitoring of both the field models contributed to the success of disease containment.

## Progression of COVID-19 in India

When the count of COVID-19 cases reached 300 by March 21, the Prime Minister of India took an intervention of nationwide lockdown. On March 24, India went into the initial phase of the lockdown of 21 days.

Initially, India was able to maintain a “steady phase” in the COVID-19 spread. On March 31, 2020, a religious congregation event, known as *Tablighi Jamaat*, held in Delhi appeared as a hotspot for the novel virus spread. The number of pilgrims attending this congregation was more than 9000, with the majority from different states across India, and approximately 960 missionaries were from 40 other nations.^2^ According to the Ministry of Health and Family Welfare, out of a total of 14 378 confirmed cases till April 18, 2020, 4291 were from this religious event.^3^

As there is no specific cure available for COVID-19, and the vaccines are in the early stages of distribution, containment strategy is the most viable option to stop its transmission. “A **cluster containment strategy** is containing the disease within a defined geographic area by early detection of cases, breaking the chain of transmission and thus preventing its spread to new areas.”^4^ But this demands meticulous planning and effective implementation.

## Successful Models to Contain COVID-19: Kerala and Bhilwara

In 2018, Kerala had dealt with an outbreak of Nipah, a virus responsible for brain damage, originated from bats and transmitted to human beings. Kerala was broadly stuck to the protocol and guidelines of the Indian Council of Medical Research (ICMR) but also brought its own strategy of developing a meticulous surveillance network, which they have already been in sync with during the Nipah virus outbreak traced back to 2018 and 2019.^5^ Kerala has remarkably used its experience of successfully controlling Nipah virus in 2018 to contain the COVID-19 spread after a rather uneven start. Despite being the first Indian state to report COVID-19 patients and topping the list of positive cases once, the state successfully managed to flatten the curve. Out of a total of 376 cases, 179 recoveries and 3 deaths have been reported. By the end of April, active cases remained at 176.^6^

The immediate steps taken by the district administration are commendable. With the vision of immense contact tracing and self-isolation, containment teams used a map and flowchart to list the places and people who could have been their contact. With the help of social media, the flow of information took place smoothly and people were asked to dial a hotline for assistance.

Kerala used massive testing, extensive contact tracing, and community mobilization for containing the virus and keeping the mortality rate low. Kerala’s long-term investment into its health care system, higher literacy rates, and exposure to previous epidemics prepared it well to flatten the curve of the COVID-19 crisis with strict enforcement of 28 days of home quarantine, that is, double the incubation period of the virus.

From early March, all international passengers were screened by the state authority. The chances of skipping airport screening were minimal as they had a well-planned system to deal with this. They formulated village committees to keep the health department updated about new arrivals and ensure their home isolation. In hotspots of Kasaragod and Kannur Districts, some village *panchayats* (the representative body of a village) even launched call centers for connecting the authorities with quarantined individuals.^7^

Amidst an increase in positive cases in Kannur, the Government of Kerala implemented the “triple lock containment” strategy to check the virus spread. The strategy had helped in arresting the infection from getting spread in the neighbouring district of Kasaragod, one of the first COVID-19 hotspots in India.

The triple lock strategy, in the District of Kannur, restricted the movement of people in 3 stages, which involved a combination of technology along with human surveillance. The strict enforcement of lockdown and optimum medical care brought the district out of the grip of the virus. Also, the GPS route maps of confirmed cases were released and people could self-report if they suspected themselves of being in contact with an infected person. The clusters under observation were managed well by Geo-mapping.

**Figure 1.**
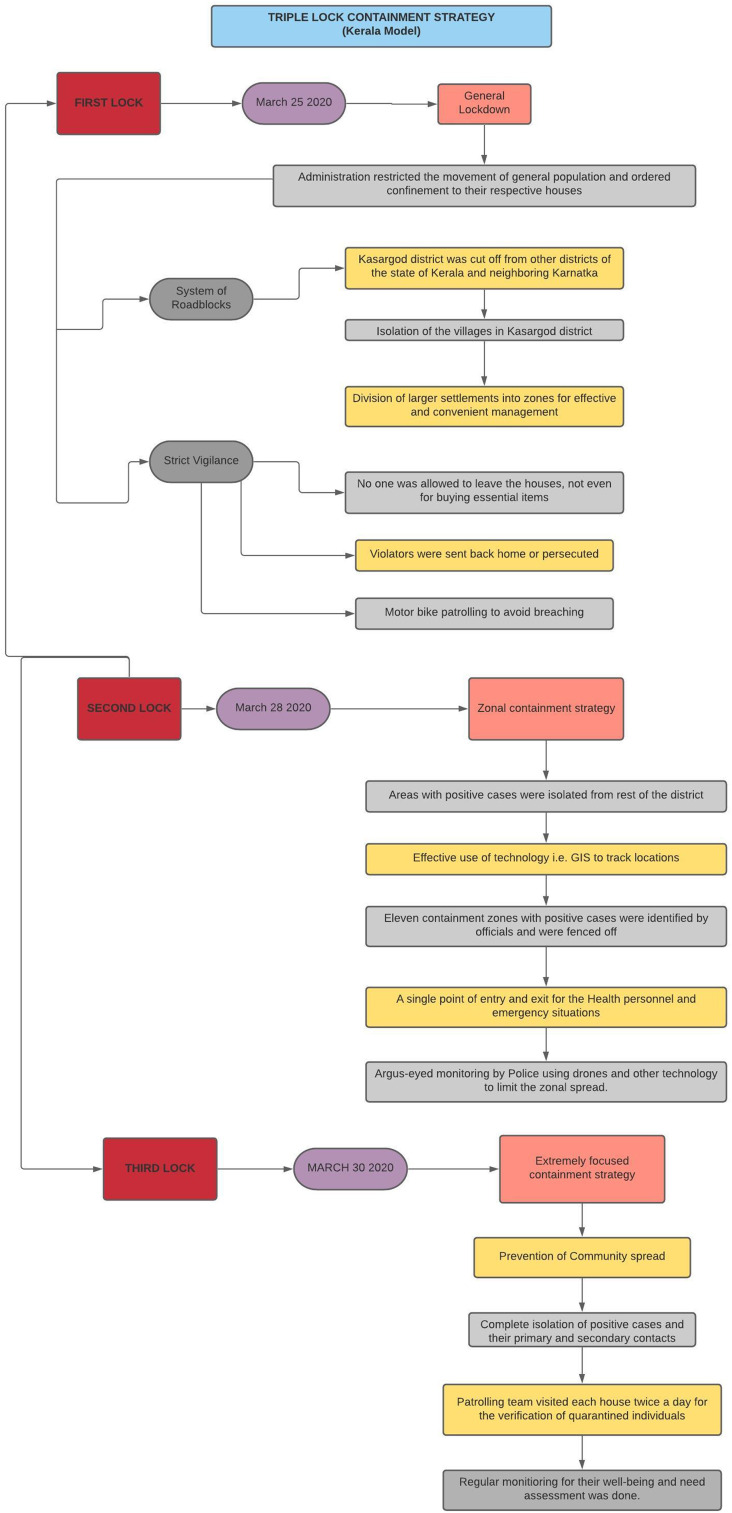
Kerala model of containment for COVID-19.

These interventions resulted in a significant drop in the reporting of new positive cases. From April 12 to April 18, only 1 positive case was reported in the state. The success estimation of this strategy can be done from the fact that there was a 97% reduction in weekly reporting of cases, that is, from 64 to barely 2 within 3 weeks^8^ (Figure 1).

A similar encouraging experience for implementation of containment strategy was reported from Bhilwara, Rajasthan. With the early emergence of coronavirus in Rajasthan, it became a reason for panic for the state and the nation. Rajasthan became the first Indian state to announce a lockdown to contain this fast-spreading pandemic.

On the other hand, Bhilwara was the city where the highest number of cases was initially reported in Rajasthan. Bhilwara, famous as a “textile town,” reported its first positive case on March 19, 2020, and then was identified as a hotspot with 27 active cases, and approximately half of the total positive cases in Rajasthan were reported to be from Bhilwara. It emerged as the epicentre for COVID-19 in Rajasthan with 6 health care workers testing as positive. The source of infection was traced to a doctor in a private hospital who was tested as positive for COVID-19.^9^

Very soon, the state government acted proactively and sealed the borders of Bhilwara and Rajasthan. Screening and contact tracing were aggressive and mass awareness was spread. Four private hospitals and 1541 rooms in 27 hotels were taken over by the district administration for disease containment. These measures happened to be fruitful as the reporting of new cases turned out to be nil in the town since March 30, 2020. District-wide disinfection drives aided in the successful containment of COVID-19.

The Rajasthan Government’s approach ensured that Bhilwara reported less than 30 cases and has had no new cases since then. The district Bhilwara of Rajasthan, once being a hotspot of this deadly virus with 27 positive cases and 2 deaths, became a containment model for the rest of the country.

**Figure 2.**
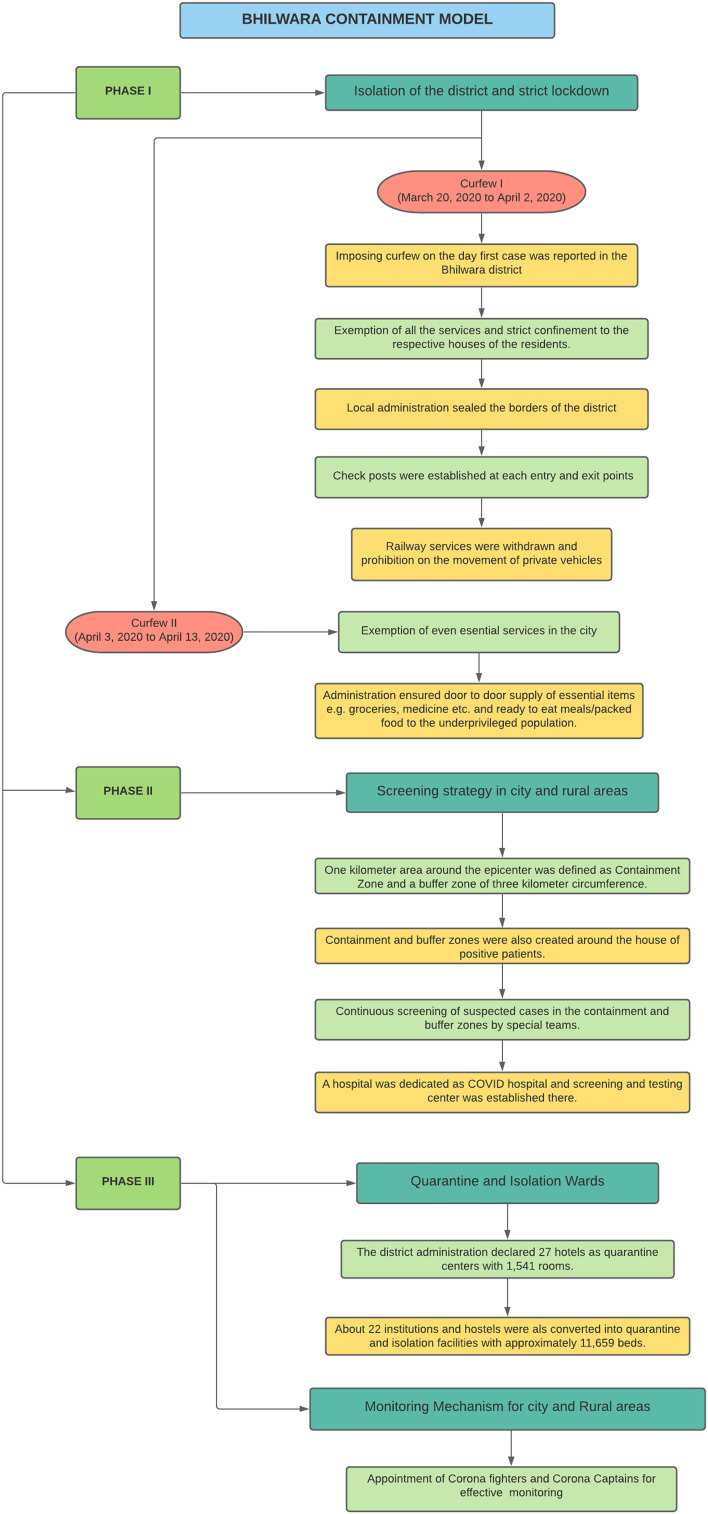
Bhilwara model of containment for COVID-19.

The Bhilwara model involved strict enforcement of a lockdown, followed by an even stricter curfew to contain the virus spread and dedicated health care and administrative team working hand in hand, with the mission of leaving no stone unturned to prevent the spread and flattening of the curve. In Bhilwara, extensive screening was done amongst more than 22 lakh people. A full lockdown backed with a curfew was enforced in the district, and the entire population was screened. The borders of the district were sealed with immediate effect (Figure 2)

The scrupulous lockdown was devoid of any relaxation, and essential items were delivered to the residents at doorsteps. As a result, the textile hub could reach zero reporting of the positive case by March 30, 2020, with 17 recoveries.^10^

## Limitations

Underreporting of cases: Due to the phobia of being quarantined or isolated, people with mild to moderate symptoms didn’t show compliance in reporting.

Under-testing: Due to a lack of testing at the appropriate time, some cases were not identified and isolated to break the chain of transmission. The test results also showed false-positive cases.

Deaths: Irrespective of the robustness of the containment strategy, the states faced a massive death toll.

## Conclusion

The containment strategy from the field of Kerala and Bhilwara can be applied to other districts of the nation, especially hotspots. There was a 76% reduction in the positive cases within 2 weeks of triple-lock containment in the District of Kasaragod, Kerala, with zero reporting of new cases from May 1, 2020, to May 11, 2020. The District of Bhilwara reported a reduction in the frequency of cases after March 30, 2020, for the first time since the outbreak. The district reported only 1 case since March 31, 2020, from being a hotspot with 27 positive cases in a single day.

Kerala implemented the strategy of triple-lock containment with emphasis on the use of technology, for example, GIS and drones, for monitoring purpose along with motorcycle patrolling, whereas the Bhilwara model focused on the phase-wise implementation of curfew/lockdown, screening strategy, and quarantine/isolation facilities followed by monitoring mechanisms that consisted of an appointment of corona fighters and corona captains in the district. Both models appeared as a boon amidst the surge of COVID-19 positive cases nationwide and can be adopted to reduce the count and maintain the flattened epidemiological curve.
